# A Comprehensive Overview of Postbiotics with a Special Focus on Discovery Techniques and Clinical Applications

**DOI:** 10.3390/foods13182937

**Published:** 2024-09-17

**Authors:** Anand Kumar, Katelyn M. Green, Manmeet Rawat

**Affiliations:** 1 Biochemistry and Biotechnology Group, Los Alamos National Laboratory, Bioscience Division, Los Alamos, NM 87545, USA; kmgreen@lanl.gov; 2 Department of Medicine, The Penn State University College of Medicine, Hershey, PA 17033, USA; mrawat@pennstatehealth.psu.edu

**Keywords:** postbiotics, microbial metabolomics, discovery, approaches, challenges, clinical application, ongoing trials

## Abstract

The increasing interest in postbiotics, a term gaining recognition alongside probiotics and prebiotics, aligns with a growing number of clinical trials demonstrating positive outcomes for specific conditions. Postbiotics present several advantages, including safety, extended shelf life, ease of administration, absence of risk, and patentability, making them more appealing than probiotics alone. This review covers various aspects, starting with an introduction, terminology, classification of postbiotics, and brief mechanisms of action. It emphasizes microbial metabolomics as the initial step in discovering novel postbiotics. Commonly employed techniques such as NMR, GC-MS, and LC-MS are briefly outlined, along with their application principles and limitations in microbial metabolomics. The review also examines existing research where these techniques were used to identify, isolate, and characterize postbiotics derived from different microbial sources. The discovery section concludes by highlighting challenges and future directions to enhance postbiotic discovery. In the second half of the review, we delve deeper into numerous published postbiotic clinical trials to date. We provide brief overviews of system-specific trial applications, their objectives, the postbiotics tested, and their outcomes. The review concludes by highlighting ongoing applications of postbiotics in extended clinical trials, offering a comprehensive overview of the current landscape in this evolving field.

## 1. Introduction

With the increasing recognition of the microbiota’s role in health and disease, there is a growing need to explore alternative avenues for maintaining a healthy microbiota, linked to enhanced productivity and reproductive efficiency [1]. Earlier attempts to categorize a healthy or core microbiota in humans were unsuccessful [2,3], revealing that it is not the core or unique key species of microbes that influence health or disease states [4,5]. Instead, it is the conserved functional redundancies of microbial species that significantly vary among individuals. Furthermore, the recognized diversity of the microbiome does not necessarily reflect the host’s healthy or beneficial status; rather, optimal performance is dictated by age and sex [6,7]. A healthy microbiota acts as a buffer within the microbial community, resisting external and internal insults such as antibiotics, infection, and stress by promptly reverting from a perturbed state (dysbiosis) to a homeostatic state (eubiosis).

**Figure 1:**
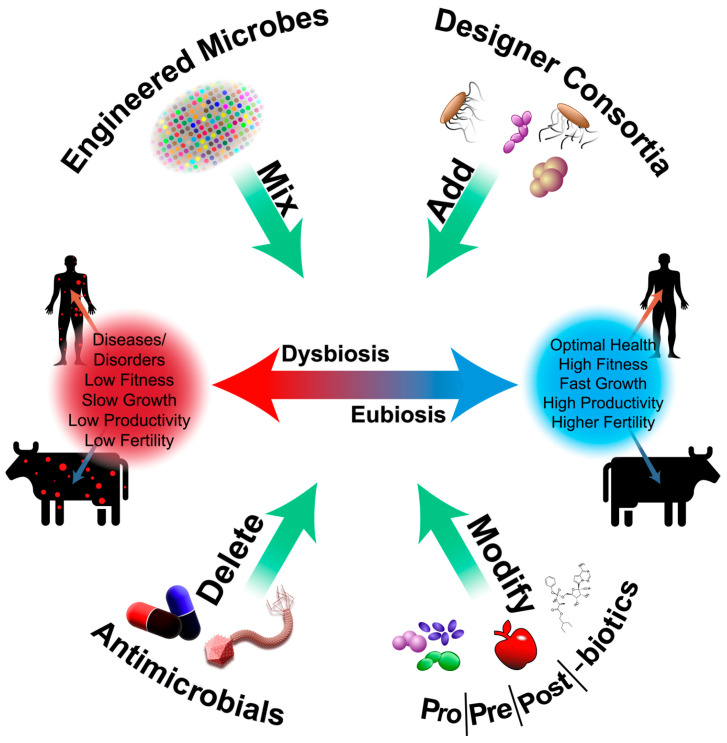
Overview of four broadly followed approaches to transition from a dysbiotic state to a eubiotic state, aiming to enhance the health, fitness, and growth productivity of both humans and animals.

Broadly, four approaches, as depicted in Figure 1, have been pursued, with the most recent addition being postbiotics, paralleling the prebiotic concept. (1) **Addition:** In this approach, a group of microbial species, either defined and tailored or undefined (like fecal transplant), is introduced to the dysbiotic state to correct underlying disease conditions (e.g., *C. difficile*). This approach induces rapid changes in the abundance and composition of the existing dysbiotic flora. (2) **Modification:** Agents such as probiotics, prebiotics, or postbiotics are supplemented to correct the dysbiotic state and improve overall health. These agents modify the existing microflora by enhancing beneficial bacterial populations. The effect on transitioning to a eubiotic state is subtle when using a single agent but can have a more moderate influence when combined. (3) **Deletion:** This approach involves the selective depletion of harmful pathogens present in a perturbed microbial community. It employs narrow-spectrum antibiotics targeting pathogens or uses phage therapy against known pathogens. The hope is that by selectively removing the main culprit pathogen, the host returns to a healthy microbiota state. This approach brings subtle changes to microbiota correction and depends on how the host responds to treatment. (4) **Engineering Microbes:** This approach leverages synthetic biology to engineer microbes that can sense and respond to changes in conditions. Some studies have highlighted the engineering of microbes to produce anti-inflammatory molecules in inflammatory conditions by sensing inflammation or continuously producing these molecules upon introduction. The ability to revert back to a eubiotic state is subtle, similar to the other two approaches. In summary, Figure 1 illustrates four diverse strategies, each with its unique mechanisms, to restore microbial balance and promote overall well-being. 

While there is no universally accepted definition of postbiotics, the terminology is rooted in the probiotic field. The International Scientific Association of Probiotics and Prebiotics (ISAPP) defined postbiotics as the “preparation of inanimate microorganisms and/or their components that confers a health benefit on the host” [8]. A recent review article defines the concept of postbiotics in simple terms as “intact non-viable microbes or cell fragments, with or without metabolites, that provide a health benefit”. The article particularly emphasizes that purified metabolites should not be classified under the term postbiotics [9]. Prebiotics are complex carbohydrates like inulin derivatives, lactulose, milk oligosaccharides, short-chain galacto-oligosaccharides, and long-chain fructo-oligosaccharides and serve as food for probiotics. Postbiotics are metabolites or byproducts of probiotics and/or gut bacterial metabolites. In essence, prebiotics are selectively consumed by probiotics to produce bioactive metabolites known as ‘postbiotics’. Table 1 highlights the distinctive characteristics associated with the usage of each of these words.

**Figure 2:**
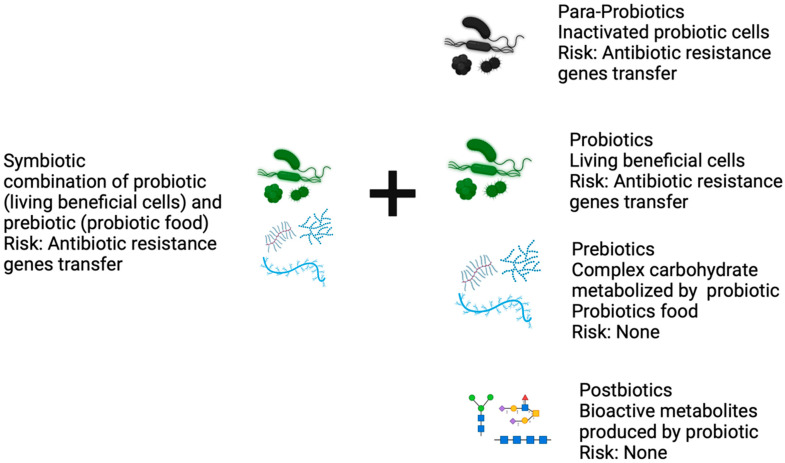
Ingredients of probiotics, prebiotics, symbiotics, and postbiotics. This illustration was generated using the BioRender tool.

Recently, postbiotics have garnered immense interest due to their added benefits compared to probiotics and prebiotics (see Figure 2). According to a report published by Global Market Insight, the postbiotic global market was valued at USD 11.6 million in 2023 and is projected to grow at a compound annual growth rate (CAGR) of approximately 11.3% from 2024 to 2032, reaching an estimated USD 30.2 million by 2032. The majority of postbiotic supplements are sold in liquid form, with North America representing the largest regional market, accounting for over 30%, driven by increasing consumer awareness about gut health [10]. Currently, both thermal methods (such as pasteurization, sterilization, and ohmic heating) and non-thermal methods (including pulsed electric field (PEF), ultrasound (US), irradiation, and supercritical carbon dioxide) are used to produce postbiotics [11]. Overall compared to probiotics, postbiotics are safer [12,13,14], generally possess a longer shelf life, are easily producible, pose no risk of antibiotic resistance gene transfer, can be administered to individuals with immunocompromised conditions, are easy to encapsulate, can be delivered to specific sites [15], have similar and/or better beneficial effects to probiotics [16], are readily patentable [8], and can undergo trials using existing standard pharmacological guidelines by documenting pharmacodynamics and kinetics. It is worth noting that symbiotics, initially conceived as a combination of both probiotics and prebiotics, have now been defined as “a mixture comprising live microorganisms and substrate(s) selectively utilized by host microorganisms that confers a health benefit on the host” [17]. Symbiotics might have some advantages compared to prebiotics, probiotics, or postbiotics, as in addition to probiotic beneficial effects, the metabolism of prebiotics by probiotics also confirms the added beneficial effects. While postbiotics are bioactive molecules that probiotics partially rely on to bring about beneficial effects on the host, postbiotics provide direct benefit to the host irrespective of the specific probiotic species/strain and/or prebiotic activities [18,19,20,21]. In the following sections, the authors endeavored to review postbiotic terminology and classifications, brief mechanisms of action, methods for discovering postbiotics, the initial steps in postbiotic discovery through microbial metabolomics, commonly employed methodologies, and the limitations in discovering and characterizing postbiotics. The authors also discuss the challenges and prospects in postbiotic discovery, as well as completed clinical trials of postbiotics and ongoing clinical trials registered in ClinicalTrials.gov. The authors aim to capture readers’ attention in the buzz surrounding the term “postbiotic” and the challenges in discovering postbiotics.

**Table 1:** This table clarifies and compares the distinctiveness of commonly used terminologies in the field. It outlines their definitions as per ISAPP, along with examples, safety concerns, patentability, shelf life, and manufacturing considerations.

	Probiotics	Prebiotics	Symbiotics	Parabiotics	Postbiotics
Associated synonyms	Beneficial bacteria	Food for probiotics	Combination of pro- and prebiotics	Killed or inactivated probiotic	Metabiotics, postmetabolites, paraprobiotic, ghost probiotics
Recent definition per ISAPP	Live microorganisms that, when administrated in adequate amounts, confer a health benefit on the host	A substrate that is selectively utilized by host microorganisms, conferring a health benefit	A mixture comprising live microorganisms and substrate(s) selectively utilized by host microorganisms that confers a health benefit on the host	Inactivated microbial cells (non-viable) that confer a health benefit to the consumer	Preparation of inanimate microorganisms and/or their components that confers a health benefit on the host
Examples	*Lactobacillus*, *Bifidobacterium*, *Saccharomyces*, etc.	Inulin, long-chain, short-chain oligosaccharides, etc.	*Lactobacillus*/*Bifidobacterium*/*Saccharomyces* + inulin, etc.	Dead or inactivated probiotics cells and teichoic acid, exopolysaccharides, fimbria, chitin, pili, etc.	Lysate, cell-free supernatant, SCFAs, peptides, bioactive molecules, urolith A, teichoic acid, exopolysaccharides
Antibiotic resistance risk and safety concerns	Present	Absent	Present	Present	Absent
Patentability	Live organisms patent not possible	Possible	Possible	Possible	Easy
Shelf life	Limited	Long time	Limited	Long time	Long time
Manufacturing quality control	Challenging	Relatively easy	Challenging	Relatively easy	Relatively easy

Note: Researchers frequently use the terms “postbiotics” and “parabiotics” interchangeably, though some make distinctions in their meanings [22].

## 2. Postbiotics

### 2.1. Terminology and Classification

The term postbiotic was derived from ‘post’, meaning ‘after’, and ‘biotic’, relating to or resulting from living organisms, to suggest ‘after life’. Postbiotics can also be referred to as metabiotics, postmetabolites [23], paraprobiotics, or ghost probiotics [24]. Postbiotics are bioactive substances such as enzymes, peptides, teichoic acids, cell lysate, microbial fractions—peptidoglycan-derived muropeptides, polysaccharides, cell surface proteins, organic acids, etc., that are produced by or released during microbial activity and known to have a positive impact on the host’s gut health and beyond [25,26,27,28]. Bacteria generate these bioactive metabolites throughout their life cycles to regulate their growth, development, reproduction, and cell–cell (bacterial–bacterial, bacterial–viral, bacterial–fungal, and bacterial–host) communication. Additionally, these metabolites promote the growth of beneficial bacteria and offer protection against diverse external and internal stressors [29,30]. When released into the local environment, these metabolites also influence the host’s cellular, metabolic processes, and physiological functions. However, the strict definition of a metabolite is “a product of biochemical reactions, with molecular weights less than 1500 Da, which exists inside (predominantly intracellular) and outside (extracellular) in the eukaryotic or prokaryotic cell” [31]. Therefore, postbiotic terminology theoretically encompasses both bacterial metabolites (low molecular weight < 1.5 kDa) and other macromolecular (high molecular weight > 30 kDa) structures.

Postbiotics are classified based on various factors. Based on molecular weight, they are classified as high-molecular-weight and low-molecular-weight postbiotics. On a structural basis, they are classified as peptides, teichoic acids, and plasmalogens [32]; on a composition basis, they are classified as carbohydrates (e.g., teichoic acids and galactose-rich polysaccharides), proteins (e.g., p40, p75 molecule, lactocepin), lipids (e.g., butyrate, acetate, propionate, lactate, dimethyl acetyl-derived plasmalogen), vitamins (e.g., B-group vitamins, organic acids—3-phenyllactic acid and propionic), and other complex molecules (e.g., lipoteichoic acids, peptidoglycan-derived muropeptides) [28]. Finally, based on their physiological function, these are classified as having anti-obesogenic, antioxidant, anti-inflammatory, hypocholesterolemic, antihypertensive, and anti-proliferative effects and exhibiting immunomodulatory properties [28].

### 2.2. Postbiotics’ Mechanism of Action 

**Figure 3:**
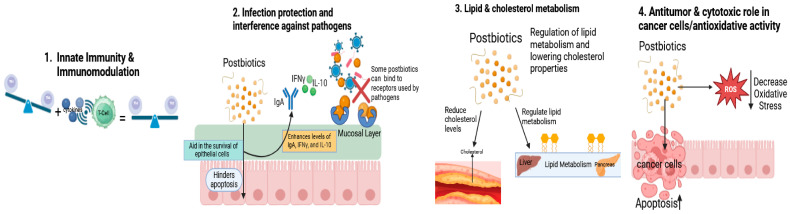
The four demonstrated main mechanisms of action of postbiotics. This illustration was generated using the BioRender tool.

Postbiotics provide various mechanisms of action through influencing gut host health, gut microbiota, nutrition, and immune response (Figure 3). Postbiotic agents hold promise for therapeutic benefits by interfering with pathogen establishment while maintaining barrier function of the intestinal mucosa. This enhances the host–microbiome balance, which therefore likely to contribute to a eubiosis state [33,34]. Postbiotics can control the immune and nervous systems via specific cellular and molecular mechanisms [35]. They also promote innate immunity, reduce pathogen-induced inflammation, and aid in the survival of epithelial cells in the intestine [33]. Immunomodulation, infection prevention, lipid/cholesterol metabolism, and anti-tumor/antioxidative activity are a few well-documented properties of postbiotics [36]. Postbiotics can restore the imbalance between Th1 and Th2 lymphocytes, which is crucial for immune regulation. Due to their immunomodulatory properties, postbiotics are being utilized as adjuvants in the treatment and prevention of COVID-19 in patients [37]. Postbiotics mediate antibacterial activities by affecting molecular structures of pathogen-binding receptors present on enterocytes and sometimes compete for the same receptors that pathogens bind to [36]. Postbiotics also play a role in lipid metabolism and reduce incidents related to the cardiovascular system [36]. Postbiotics modulate native probiotic strains, aiding the maintenance of the intestinal microbiota and host homeostasis [38]. Postbiotics can exploit bacterial extracellular vesicle secretion systems to influence microbe–microbe communication and host signaling pathways [39], though the detailed mechanisms have yet to be fully explored. 

Probiotic-derived molecules have been shown to slow down the formation of tumors and the progression of cancer but not affect healthy intestinal cells [40]. Postbiotics play a key role by mediating the communication between the mutualistic microbiota and the immune system, which in turn can govern immune system activity to maintain homeostasis and promote beneficial health [41]. Postbiotics also play a cytotoxic role against cancer cells and increase apoptosis in these cells. In fact, p40 is a protein that is soluble and is produced by *Lactobacillus rhamnosus* GG and *Bacteriodes fragilis*, provide agility for the epithelium in the intestine by inhibiting apoptosis in epithelial cells and increasing the levels of IgA, IFN-γ, and IL-10 [41,42]. IL-10 is critical for a proper balance of inflammatory and immunopathologic responses and is considered an inhibitory cytokine; some postbiotics increase the levels of IL-10 [43]. IFN-γ plays a critical role in developing innate and adaptive immune responses and is an important cytokine in promoting pathologic inflammatory processes [43,44]. 

The ISAPP convened a panel of experts to define the scope of postbiotics and identify potential mechanisms of action. The panel suggested the top five mechanisms of action as follows: (1) modulation of the resident microbiota; (2) enhancement of epithelial barrier functions; (3) modulation of local and systemic immune responses; (4) modulation of metabolic responses; and (5) systemic signaling via the nervous system [45]. Postbiotics are a mixture of various active substances, and their effects are mediated by a wide variety of mechanisms. Studies have reported postbiotics having positive effects on reducing blood pressure, cholesterol levels, and even body weight. Modulation of the resident microbiota through molecules present in postbiotics shows antimicrobial activity. These molecules can also modulate bacteria indirectly through quorum sensing, cross-feeding, and adhesions [45]. Modulation of systemic immune responses like indole derivatives, histamine, keto acids and SCFAs (short-chain fatty acids) allows for the interaction of microorganism-associated molecular patterns [45]. 

Postbiotics also exert their effects through various mechanisms involving peptides, enzymes, SCFAs, vitamins, teichoic acid, and plasmalogens [46]. Peptides inhibit cell wall synthesis and have antimicrobial properties affecting the brain, and intestines. Enzymes can degrade polymeric substances and eradicate biofilms, decreasing reactive oxygen species (ROS) and impacting the skin, intestines, and pancreas. SCFAs serve as an energy source for epithelial cells, influencing the colon and liver. Vitamins are crucial for the absorption of colonic thiamine and help free riboflavin, affecting the heart and colon. Teichoic acid plays a vital role in antibiotic resistance and anti-tumor activity, impacting bone marrow and dendritic cells [46]. Postbiotics can indirectly contribute to wound healing inside the gut [47], as also shown in a study where *L. brevis* enhanced epithelial barrier integrity by improving platelet accumulation at the wound site [48]. Other studies have demonstrated that postbiotics positively affect host metabolism and signaling pathways through microbial-derived vitamins and SCFAs, which can act as metabolic modulators. For example, propionate can alter lipid metabolism and increase insulin sensitivity and glucose tolerance [49]. Although the precise mechanisms of action have yet to be established, postbiotics have been shown to positively influence intestinal ailments, respiratory conditions, alcohol-induced liver disease, skin conditions, and dental caries [50].

Overall, postbiotics have mechanisms of action, both locally and systemically, that support epithelial cell survival and play a key role in facilitating communication between certain bacteria and the immune system, helping to maintain equilibrium and promote beneficial health [28]. With increasing research interest in postbiotics, it is likely that various applications in human health and their underlying mechanisms of action will be uncovered.

### 2.3. Navigating the Postbiotic Discovery Landscape

**Figure 4:**
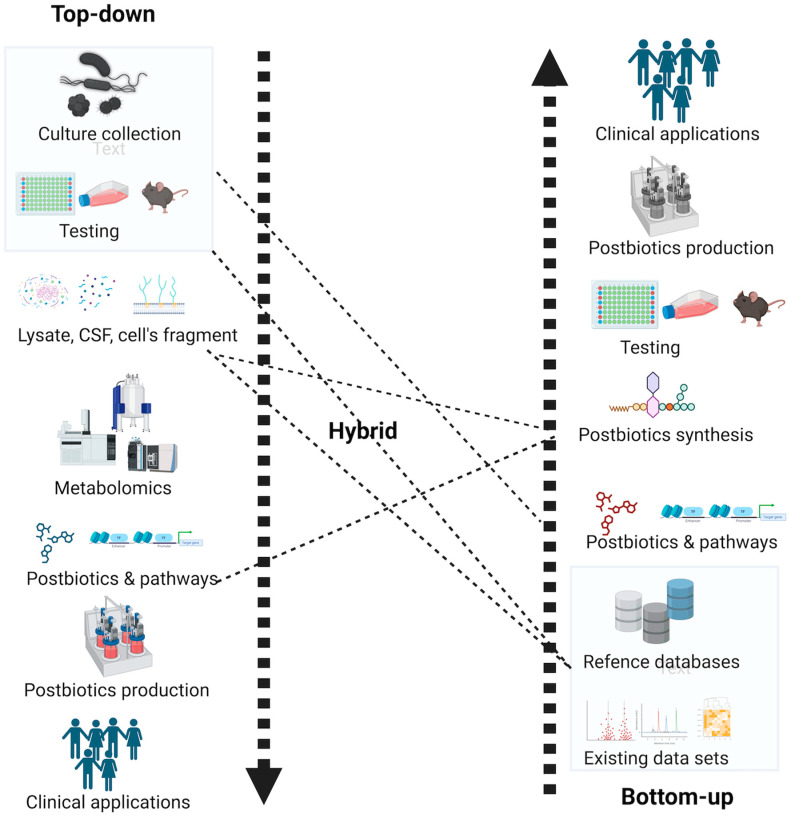
Overview of commonly employed approaches for discovering postbiotics, with a process that mirrors the identification of new metabolites or molecules. This illustration was generated using the BioRender tool.

The postbiotic discovery follows a dual-pathway approach for discovering antimicrobial or anti-cancerous compounds, employing three main methods (Figure 4): top-down, bottom-up, and hybrid approaches. In the top-down approach, species and strain collections with known phenotypic effects are screened in in vitro or in vivo models [51]. This is followed by the exploration of their lysate, extract, or cell-free supernatant (CFS) to identify bioactive metabolites using established methods, with the subsequent step involving understanding their gene regulatory pathways. However, a major drawback of this approach is the necessity for an extensive and diverse culture collection of microbes, along with the development of high-throughput phenotypic screening assays [52]. Challenges arise in culturing all microbes in a lab setting, and screening codependent microbes for their effects becomes impractical [53]. Additionally, developing assays requires prior knowledge of expected functions and expertise in synthetic biology.

On the other hand, the bottom-up approach, also known as the genome mining approach, involves in silico analysis of metabolite molecules with the potential for expected phenotypic effects. It begins by exploiting existing metabolite databases to predict their potential functions, followed by testing the predicted metabolite functions on the host in vitro and in vivo models [54,55]. While this approach overcomes challenges associated with the top-down approach, it does have limitations, including the requirement for extensive metabolite databases, lack of characterization for many metabolites, the need for sophisticated tool development (such as Artificial Intelligence (AI) or Machine Learning (ML)-based tools) often based on limited or hypothesized knowledge, difficulty in connecting to regulatory gene networks, and the fact that predictions may not necessarily correspond to known phenotypic effects.

The hybrid approach combines the merits associated with top-down and bottom-up approaches, offering flexibility based on resource availability and specific research needs. In theory, this approach is considered ideal for exploring, identifying, and isolating novel classes of postbiotics. However, in application, the main limitation is the requirement for truly interdisciplinary research efforts and logistical support, which can be challenging to find in conventional research institutions.

Regardless of the approach followed, the ultimate goal is to either produce such bioactive postbiotics commercially through synthesis mechanisms or, if not possible, to genetically overexpress such molecules in homologous or heterologous bacterial species. Furthermore, the fermentation behavior of probiotic species and strains significantly impacts the quality and quantity of postbiotics [56]. Therefore, it is imperative to thoroughly discover and fully characterize these postbiotics before increasing production.

#### 2.3.1. Microbial Metabolomics—The First Step in Postbiotic Discovery

In the preceding section, we discussed the classification of postbiotics based on various factors. However, from a discovery perspective, a relevant classification of postbiotics is based on their molecular weight—either low or high. This classification proves instrumental in describing discovery techniques, which are tailored according to molecular weight considerations. While a comprehensive overview of these methods is beyond the scope of this review, we will briefly outline their applications and limitations to familiarize the reader with the available methodologies.

Metabolomics, especially in microbial contexts, lags behind other omics techniques in terms of evolution and application. This gap is primarily attributed to the complex nature of metabolites produced and the absence of a comprehensive reference database, with approximately 80–90% of discovered metabolites remaining unidentified [57,58]. Updating this database is certainly a gradual process, hindered by cost constraints and logistical challenges associated with conducting metabolomic studies on existing collections of microbes. A parallel can be drawn with the Human Genome Project—massive funding from government agencies could expedite the metabolite database’s rapid expansion. It is noteworthy that metabolite profiles can vary depending on growth conditions, gene expression, and protein expression. Promisingly, ongoing efforts involve the application of ML- and AI-based tools to facilitate de novo metabolite classification and characterization.

Microbial metabolomics, a swiftly progressing field within systems biology, is poised to provide detailed insights into the true physiological state of microbes. Metabolites, functioning as end signals, play a pivotal role in connecting observed microbial phenotypes. The sequence of events initiated by internal and/or external stimuli involves specific gene expression, followed by functional protein expression, culminating in the production of metabolites that contribute to the observed phenotype. Consequently, the information gleaned becomes more valuable when integrated with other omics methods such as transcriptomics and proteomics, offering a comprehensive understanding of the molecular networks at play. Broadly, two types of metabolomics are being performed [59]: (1) Untargeted metabolomics involves a broad and thorough analysis that encompasses the measurement of all metabolites in a given sample, including those that are unidentified or unknown. (2) Targeted metabolomics focuses specifically on measuring a predetermined set of well-characterized and biochemically annotated analytes. Untargeted metabolomics establishes foundations for discovery and hypothesis generation, while targeted metabolomics is hypothesis-driven and often applied for the validation of previously identified processes by leveraging a priori knowledge of metabolic pathways and mechanisms [59]. Further based on metabolite chemical classes and physical properties, the metabolome can be further classified into the proteome and lipidome [60]. Although a few reviews have specifically discussed microbial metabolomics in depth [61,62,63,64], none of the reviews were focused on the postbiotic discovery context, which is the goal of the sections below.

In microbial metabolomics, produced metabolites are analyzed both qualitatively and quantitatively. The most commonly employed technique is mass spectrometry (MS), often combined with other separation techniques such as gas chromatography–mass spectrometry (GC–MS) and liquid chromatography–mass spectrometry (LC-MS). Nuclear magnetic resonance (NMR) is the next most commonly employed technique. Recently, researchers have also started using multiple techniques to decode microbial metabolites [61]. One has to understand that the choice of employed technique is often dependent on the experimental objective and sample type/matrix being investigated [65]. In the below sections, we describe each of these techniques’ applications and limitations in the context of postbiotics.

#### 2.3.2. NMR—Application and Limitations in Postbiotic Discovery

NMR uses a strong magnetic field to probe the intrinsic properties of atomic nuclei, measuring the nuclei of molecules through specific spectroscopy using isotopes such as ^1^H and ^13^C. Hence, this technique can be applied to study the kinetic and structural properties of solids, liquids, and gases [66,67]. In addition to X-ray crystallography, NMR provides a time series kinetic information of metabolites, which is difficult to decode by crystallography [68]. In detecting microbial metabolomics, NMR is highly reproducible, and by integrating proton NMR signals, identified metabolites can be quantified. In comparison to MS-based techniques (LC-MS and GC-MS), the NMR technique is a good fit for analyzing compounds that pose challenges in ionization, which is a prerequisite for MS-based techniques. Note that in the below section, the probiotic genera name “*Lactobacillus*” has been amended as per a recent reclassification effort proposed by Zhang et al. [69]; hence, it is not likely to correspond to published article genera names. 

In a recent study, the authors utilized ^1^H NMR spectroscopic analysis to examine purified lipoteichoic acid (LTA), a surface component found in Gram-positive probiotic lactobacilli strains—specifically *Lactiplantibacillus plantarum* MTCC 5690 and *Limosilactobacillus fermentum* MTCC 5689, as well as the commonly used strain *L. rhamnosus* GG (LGG) [70]. Since LTA functions as a postbiotic and serves as a bacterial microbe-associated molecular pattern (MAMP), interacting with the pattern recognition receptors (PRRs) of host cells in what is known as MAMP-PRR interactions, it plays a crucial role in activating various signaling cascades within the host. To investigate the species- and strain-specific effects of LTA, the authors conducted comparisons in both in vitro and in vivo models. Notably, the *L. rhamonus* strain exhibited a lower level of alanylation on its LTA compared to other strains. Consequently, this led to differential inflammatory effects observed in the in vitro and in vivo models, where *L. rhamonus*’s LTA demonstrated heightened anti-inflammatory properties [70].

In another study, researchers aimed to pinpoint anti-biofilm postbiotics within the cell-free supernatant (CFS) of the probiotic strain *Limosilactobacillus reuteri* DSM 17938 [71]. The authors conducted a ^1^H-NMR metabolomic analysis of both the CFS and its sub-fractions (SurE 10 K with a molecular weight <10 kDa) to discern and quantify various postbiotic compounds. The NMR analysis successfully identified and quantified several compounds, predominantly organic acids and amino acids, with lactate emerging as the most abundant metabolite in both CFS and SurE 10K samples. Notably, formate and glycine were exclusive to the CFS, setting them apart from the SurE 10K samples [71].

To investigate the extracellular vesicles (EVs) derived from specific *Lactobacillus* strains with either inhibitory (*Lactobacillus crispatus* BC3, *Lactobacillus. gasseri* BC12) or non-inhibitory (*L. crispatus* BC5, *L. gasseri* BC13) effects on HIV-1 replication, researchers conducted ^1^H-NMR metabolomics [72]. The aim was to identify metabolites/postbiotics associated with the EVs of these probiotic strains. The study revealed 42 molecules, primarily falling into classes such as organic acids, amino acids, sugars, and nitrogen bases. Interestingly, five of these metabolites showed a significant correlation with the antiviral activity. The EVs with inhibitory effects (*L. crispatus* BC3 and *L. gasseri* BC12) were notably linked to high concentrations of methionine, glycine, hypoxanthine, and glutamate. In contrast, non-inhibitory EVs (*L. crispatus* BC5 and *L. gasseri* BC13) were characterized by the predominant presence of asparagine [72].

The major limitations of this method to characterize postbiotics are as follows: NMR necessitates the derivatization of the analyzed compound and requires exceptionally high input concentrations of metabolites [73], and it has limited sensitivity and separation capacity. Therefore, the complexity of microbial metabolite compositions and concentrations, ranging from pmol/L to mmol/L, restricts the widespread application of the NMR technique in microbial metabolomics [74]. Moreover, the availability of quantitative methods and commercial software for NMR data analysis is often limited. Recent advancements in magnet technology, particularly the development of permanent magnets, cryogen-free superconducting magnets, and hyperpolarization technologies, have significantly improved the convenience, cost-effectiveness, and sensitivity of NMR [74]. 

In summary, NMR instruments are relatively costly compared to MS, and require highly skilled operators and substantial laboratory space, including nonvibrational floors and protection from magnetic and radio-frequency interference. These factors, combined with the overarching challenge of low sensitivity, have posed difficulties for NMR in broadening its user base in microbial metabolomics [74].

#### 2.3.3. Exploring Applications and Limitations of GC-MS in Postbiotic Discovery

MS is a versatile analytical technique employed for both qualitative and quantitative analysis. It facilitates the identification and quantification of a broad range of analytes by measuring their mass-to-charge ratio (*m*/*z*), where ‘m’ represents the molecular weight of the ion in Daltons, and ‘z’ signifies the number of charges present on the measured molecule. Various types of mass analyzers are employed for MS analyses, including quadrupole, magnetic sector, radio-frequency ion trap, time of flight (TOF), orbitrap, and ion cyclotron resonance. These analyzers can be combined to perform tandem mass spectrometry (MS/MS), where two mass analyses occur in series, typically with a fragmentation step in between [75]. The chromatography technique exploits the differences in affinities of analytes in a mixture for the stationary and mobile phases to achieve their separation and allows for the identification and quantification of individual metabolites within a complex mixture. Hence, quite often, both methods are combined to achieve the highest sensitivity and specificity that the standalone NMR technique lacks. 

The MS process comprises three essential components:

1. **Ionization:** At this initial stage, the analyte undergoes ionization through an ionizing source.
2. **Mass Analyzer:** Ionized analytes are then sorted based on their *m*/*z* ratio.
3. **Detector:** The detector measures the ions and presents their mass spectrum chart.

Typically, the sample analyte exists in either a liquid or gaseous phase. Consequently, GC and LC techniques are commonly integrated with MS to detect molecules. For solid analyte samples, various methods such as digestion in strong and hot acids can transform them into a liquid phase. Alternatively, laser ablation, spark ablation, or electrothermal vaporization can convert them into a gaseous (aerosol) phase. Therefore, solid-, liquid-, and gas-phase samples can be effectively analyzed using GC-MS or LC-MS techniques.

In a comprehensive comparative study, researchers scrutinized the volatile compounds, organic acid composition, and antioxidant and antimicrobial activities of postbiotics derived from six distinct probiotic strains (*Lactiplantibacillus plantarum* RG11, RG14, RI11, RS5, TL1, and UL4) [76]. These strains demonstrated inhibitory effects against *Pediococcus acidilactici* 446, *Escherichia coli* E-30, *Salmonella enterica* CS3, and *vancomycin-resistant Enterococci*. The analysis was carried out using GC-MS. The study findings underscored positive correlations between the production of organic acids (including acetic acid, lactic acid, ascorbic acid, and caproic acid) and inhibitory activity against the specified pathogens as well as antioxidant activity. The authors concluded that the composition and functional attributes of postbiotics, originating from the six *L. plantarum* strains, were contingent on the specific strain and significantly influenced by the fermentation medium employed [76].

In a separate investigation, researchers delved into the anti-*Staphylococcus* properties of postbiotics extracted from *Lactobacillus acidophilus*, *Lacticaseibacillus paracasei*, and *L. plantarum* through in vitro experiments [77]. The chemical composition of these postbiotics was meticulously identified using GC-MS. The results indicated that the antibacterial effects of the postbiotics were primarily attributed to compounds such as lactic acid, laurostearic acid, and isopropylidene-3,3-dimethyl. Notably, these postbiotics demonstrated robust antioxidant activities. Of particular significance, the postbiotics derived from *L. plantarum* exhibited the highest antioxidant properties when compared to those from the other probiotic strains, namely *L. paracasei* and *L. acidophilus* [77]. This sheds light on the distinctive antioxidant potential inherent in postbiotics originating from different probiotic sources. 

Researchers from Iran conducted an analysis of postbiotics derived from the bioprotective culture FRESHQ^®^ developed by Chr. Hansen, Denmark [78]. This culture is claimed to contain strains specifically chosen for their ability to safeguard dairy products against spoilage by yeast and mold, as demonstrated by GC-MS analysis. The findings revealed the presence of various compounds, including fatty acids, alkanes, aldehydes, hydrocarbons, fatty acid esters, propionic acid, and notable antibacterial and antifungal substances such as 2,4-Di-tert-butyl phenol and dotriacontane. Importantly, these postbiotics exhibited concentration-dependent antimicrobial activity against a range of strains, including *Listeria monocytogenes*, *Staphylococcus aureus*, *E. coli*, *Salmonella typhimurium*, *Aspergillus flavus*, and *Penicillium citrinum* [78]. The researchers concluded that these postbiotics hold promise for the development of materials featuring antimicrobial membranes, particularly in applications related to food preservation. 

In a separate recent study, authors utilized GC × GC-MS to characterize postbiotics derived from *Limosilactobacillus fermentum* U-21, known for its antioxidant properties, and *L. fermentum* 279, which lacks antioxidant capabilities [79]. The analysis unveiled a total of 1144 metabolites in *L. fermentum* U-21, including distinctive compounds such as neurotransmitters (e.g., γ-aminobutyric acid, adrenaline, dopamine, noradrenaline, glycine, and DOPA) and immunomodulatory substances (e.g., acetic acid, propanoic acid, butanoic acid, indolelactate, indole-3-acetamide, and indole-3-acetic acid) [79].

Due to the demonstrated effectiveness of the probiotics *Bifidobacterium bifidum* IDCC 4201 and *L. plantarum* IDCC 3501 in inhibiting tyrosinase and reducing melanin synthesis (melanogenesis), a process responsible for skin pigmentation, researchers conducted a comparative metabolomics analysis using GC-MS to identify common postbiotics produced by these strains [80]. Among the eight key postbiotics identified (alanine, leucine, methionine, phenylalanine, threonine, valine, pipecolic acid, and phenyllactic acid), phenyllactic acid stood out with significant tyrosinase-inhibitory activity. This suggests that phenyllactic acid acts as a potential anti-melanogenesis agent in food and medicinal contexts [80].

In a particular study, researchers delved into the immunostimulatory effects of heat-treated *L. plantarum* LM1004 (HT-LM1004), with a specific focus on its metabolites, particularly exopolysaccharides, using GC-MS analysis [81]. The analysis revealed the presence of seven types of fatty acids, with lactobacillic acid and palmitic acid being the most abundant cellular fatty acids. The proportions of saturated fatty acids (SFAs), unsaturated fatty acids (USFAs), and cyclic fatty acids (CFAs) in HT-LM1004 were determined to be 41.42%, 20.03%, and 38.55%, respectively. Overall, postbiotics derived from HT-LM1004 were found to stimulate the expression of MAPK/AP-1 and NF-κB, leading to the release of nitric oxide and cytokines [81]. These findings suggest that these postbiotics could be harnessed for the development of various food and pharmacological products with immunostimulatory effects.

Researchers investigated OMNi BiOTiC^®^ AAD10, a commercial product from Winclove Probiotics B.V in the Netherlands, comprising 10 distinct probiotic strains: *L. acidophilus* W55 and W37, *L. paracasei* W72, *L. rhamnosus* W71, *Ligilactobacillus salivarius* W24, *L. plantarum* W62, *Enterococcus faecium* W54, *B. bifidum* W23, *B. lactis* W18, and *Bifidobacterium longum* W51 [82]. The focus of the investigation was on the postbiotic composition, particularly volatile organic compounds (VOCs), analyzed using GC-MS. The postbiotic supernatant treatment exhibited in vitro antimicrobial activity against various microorganisms, including *S. epidermidis*, *L. monocytogenes*, *Pseudomonas aeruginosa*, *E. faecium*, *Streptococcus agalactiae*, and *Candida albicans*. Upon GC-MS analysis of the supernatant, a total of 36 VOCs were identified, with 24 VOCs showing a significant correlation with the growth of probiotic strains [82].

The authors developed a postbiotic complex derived from the probiotic strains *Lactobacillus helveticus* (HY7801) and *Lactiplantibacillus lactis* (HY449). This complex demonstrated antibacterial activity against *S. aureus* and *Cutibacterium acnes*, along with notable anti-inflammatory effects [83]. By employing GC–MS for metabolic profiling of the postbiotic complex, the authors identified specific postbiotics, namely 2-hydroxyisocaproic acid, hypoxanthine, succinic acid, ornithine, and γ-aminobutyric acid, as potential contributing metabolites for the observed antibacterial and anti-inflammatory properties [83]. The authors concluded that the development of such a postbiotic complex holds promise for applications in food, cosmetics, and pharmaceutical products. These postbiotics could serve as alternative or complementary resources to probiotics, effectively contributing to the prevention of acne and skin inflammation.

To sum up, the GC-MS technique proves effective in identifying distinct postbiotics derived from probiotics, establishing a link to various observed phenotypes such as antimicrobial activity, pigment suppression, immunostimulant effects, neurotransmission modulation, and antioxidant properties. As the metabolomics reference database continues to expand, the application of this method is expected to unveil an increasing number of specific postbiotics associated with these diverse biological activities.

GC-MS technology, while powerful, has notable limitations when it comes to discovering metabolites/postbiotics, including the following:

1. **Limited Sample Type Analysis:** GC-MS is primarily designed for volatile and semi-volatile organic compounds, making it unsuitable for the analysis of inorganic compounds, biopolymers, and samples with high viscosity or water content.
2. **Limited Sensitivity to Trace Metabolites:** Despite being a highly sensitive technique, GC-MS faces challenges in detecting trace metabolites within complex sample mixtures.
3. **Interferences:** The presence of certain compounds in samples can interfere with the accuracy of analysis, potentially leading to false positive or negative detections.
4. **Limited Structural Information:** GC-MS may not provide detailed molecular structure or functional group information, limiting the depth of structural insights into the identified metabolites/postbiotics.
5. **Logistical Challenges:** The technology is associated with a high cost, requiring specialized instruments and expertise for operation. Additionally, data analysis tools and complex sample preparation steps further contribute to the logistical challenges of using GC-MS in metabolite/postbiotic discovery.

#### 2.3.4. Application and Limitation of LC-MS in Postbiotic Discovery

Next to GC-MS, LC-MS, although relatively newer, is frequently utilized for the physical separation of analytes or postbiotics in liquid samples or solutions of solid samples. It provides a high degree of sensitivity, selectivity, and accuracy in detecting microgram or even nanogram metabolites. While the core principle is the same between the two techniques, LC-MS offers advantages in terms of ease and speed of sample extraction, shorter run times, and potentially a broader range of compound analysis compared to GC–MS [84].

LC separations are categorized into different modes based on the mechanism of interaction between the analyte and the stationary phase:

1. **Partition chromatography**: This mode relies on the varying solubility and hydrophobicity of analytes in the stationary phase compared to the mobile phase.
2. **Ion-exchange chromatography**: This separates analytes based on their ionic charges.
3. **Size-exclusion chromatography**: This method exploits differences in the sizes of analyte molecules to achieve separation.
4. **Affinity chromatography**: Analytes are separated based on their ability to bond with the stationary phase.

Based on the ionization source and type, the following LC-MS interfaces are commonly employed: electrospray ionization (ESI), atmospheric pressure chemical ionization (APCI), and atmospheric pressure photoionization (APPI) [75]. The ESI interface is useful for the analysis of moderately polar and even very polar molecules, such as metabolites, xenobiotics, peptides, nucleotides, and polysaccharides. On the other hand, APCI can be used to analyze small, neutral, relatively non-polar, and thermally stable molecules, including steroids, lipids, and fat-soluble vitamins. For the analysis of neutral compounds that cannot be ionized using ESI, APPI is employed [75].

To discover postbiotics in coffee brews fermented with the probiotics *L. rhamnosus* GG and *Saccharomyces boulardii* CNCM-I745, researchers employed an untargeted LC-QTOF-MS/MS-based metabolomics approach [85]. The analysis revealed 37 different metabolites between fermentation treatments, and putatively annotated postbiotics included 2-isopropylmalate produced by *S. boulardii* and aromatic amino acid catabolites (indole-3-lactate, p-hydroxyphenyllactate, 3-phenyllactate), as well as hydroxydodecanoic acid by *L. rhamnosus* GG. These findings suggest that LC-QTOF-based untargeted metabolomics can be an effective approach to uncover postbiotics [85].

In a study, the exopolysaccharides (EPSs) secreted by six strains belonging to *Lactobacillus crispatus* (BC1, BC4, and BC5) and *L. gasseri* (BC9, BC12, and BC14) species were analyzed using LC-MS [86]. The findings indicated that D-mannose was the most abundant monosaccharide in EPS from all lactobacilli (39–52%), followed by D-glucose (11–30%) and D-galactose (8–16%). The relative abundance of D-xylose and D-fucose was very low (<1% and 3–4%, respectively). Furthermore, the two *Lactobacillus* species exhibited significant differences in the content of D-mannose, D-glucose, and D-fucose. Specifically, the relative abundances of D-mannose and D-fucose were significantly higher in EPSs from *L. crispatus* strains compared to EPSs from *L. gasseri* strains, while the relative abundance of D-glucose was higher in EPS-LG. These key differences in EPS structures were linked to the varied effects of the probiotics on tested pathogens (*E. coli*, *S. aureus*, *Enterococcus faecalis* and *E. faecium*, *Streptococcus agalactiae*) and fungi (*C. albicans* and *Candida glabrata*), including inhibition and biofilm suppression [86].

The CFS of the probiotic *Apilactobacillus kunkeei*, extracted from honey, was tested for its in vitro antibacterial (*Bacillus cereus*, *E. coli*, *S. enterica*, *S. aureus*) and antifungal (*Aspergillus flavus*, *Aspergillus niger*, *C. albicans*) activities [87]. The results demonstrated significant growth inhibition properties. To explore the presence of postbiotics in the CFS, the authors employed LC-MS. The LC-MS analysis revealed the presence of three different unannotated antibacterial peptides with molecular weights of 9183.82, 16,643.77, and 18,339.65 Da in the CFS [87]. This suggests that the LC-MS technique can be effectively utilized to uncover unannotated postbiotics and their molecular weights. 

Researchers investigated the metabolomics of the CFS of OMNi BiOTiC^®^ AAD10, a commercial probiotic product from Winclove Probiotics B.V in the Netherlands [82]. The analysis was conducted using LC-MS to detect the composition of postbiotics. A total of 122 metabolites were identified, with 98 associated with the following groups: amino acids/peptides/metabolites and nucleotides/metabolites, while 24 belonged to the following groups: fatty acids and metabolites, carbohydrates and conjugates, as well as pharmaceuticals and xenobiotics. The growth phase correlation analysis of the CFS suggested that fumaric acid, malic acid, aspartic acid, cytidine monophosphate, and orotidine increased with culture time, while phosphoserine, creatine, pantothenic acid, tryptophan, and 9,3-methyl-2-oxovaleric acid decreased [82]. This suggests that certain postbiotics may be associated with the growth phase of probiotics, and their utilization requires consideration for phase-specific applications.

Overall, LC-MS is a relatively newer analytical technique compared to GC-MS and performs exceptionally well with solid and liquid sample types. As suggested in previous studies, it can be employed to uncover species or strain-specific postbiotic profiles. Additionally, LC-MS is effective in discovering unannotated proteins and comparing the structural basis of known postbiotics, such as EPS.

The LC-MS technique, despite its advantages, comes with certain limitations:

1. **Sample Type:** It is more suitable for solid and liquid samples but may face challenges with volatile metabolite analysis.
2. **Sensitivity:** While it is generally sensitive, some postbiotics with low concentrations may not be accurately detected.
3. **Specificity:** The trade-off between speed and precision in analysis can lead to the false detection of a few metabolites.
4. **Matrix Effect:** Fluctuations in the metabolite matrix composition can occur without being detected, hence potentially yielding biased results.
5. **Logistics:** Similar to GC-MS, LC-MS uses an expensive instrument that requires regular maintenance. Skilled labor is necessary to operate the instrument and analyze the data effectively.

#### 2.3.5. Combined MS Techniques in Postbiotic Discovery

In a study involving the beneficial bacteria *Weissella cibaria* and *Weissella confusa* isolated from honey bees, which exhibited antimicrobial and antioxidant properties, researchers conducted a comprehensive analysis of the postbiotic profile using various techniques [88]. For the identification of organic acids in postbiotics, HPLC was employed. GC-MS was used for the analysis of fatty acids, while LC-MS was utilized for a comprehensive analysis of water-soluble and water-insoluble vitamins. The HPLC results indicated that lactic acid was the highest (21.38–22.54 mg/mL) organic acid in postbiotics, with succinic acid and citric acid found only in postbiotics derived from certain strains. The GC-MS results revealed the presence of stearic acid, caprylic acid, caproic acid, and butyric acid in all postbiotics, ranging from 0.06 to 0.30% concentrations. Additionally, tridecanoic acid, eicosenoic acid, and eicosadienoic acid were identified in specific postbiotic strains. LC-MS analysis suggested high concentrations of water-soluble B-complex vitamins (B1 thiamine, B2 riboflavin, B5 pantothenic acid, and B12 cobalamin) in postbiotics, ranging from 20.19 to 351.62 μg/L, while some fat-soluble vitamins (E1 and K3) were detected in postbiotics within the range of 6.99 to 75.34 μg/L. Vitamin B12 was identified as the most abundant vitamin among those present in the postbiotics [88].

### 2.4. Challenges and Prospectus in Postbiotic Discovery

Over the past few decades, increasing progress has been made in understanding the beneficial effects of postbiotics on the host, providing some mechanistic insights into their mode of action. In addition to medical applications, research has also highlighted the potential of postbiotics in improving the shelf life of dairy products [89]. While postbiotics offer numerous advantages over probiotics and prebiotics, their discovery poses a unique challenge due to the broad definition of the term, encompassing any secreted macro- or micromolecules with biological functions. Some well-known postbiotics, such as short-chain fatty acids (SCFAs), have gained attention, but the primary question remains: how can we identify novel postbiotics? Confusion among researchers arises from the use of the term “postbiotics” to describe bioactive metabolites derived from probiotic or beneficial bacteria, even though the proposed definition includes any microbial-derived metabolites. This raises the question of whether researchers should explore metabolites from other microbial species, including pathogens, in the search for postbiotic properties. To simplify matters, the authors propose reserving the term “postbiotics” for metabolites derived from probiotic, beneficial, or commensal bacterial species.

Another challenge is the discovery of probiotics themselves, as only a limited number of species and their strains are currently utilized as probiotics. In this context, researchers are actively engaged in research efforts to develop approaches for discovering the next generation of probiotics by leveraging microbe–microbe and host–microbe interactions to isolate, identify, and characterize potential novel next-generation probiotic species [90,91].

Additionally, there are limitations associated with existing techniques for discovering metabolites, as discussed in previous sections. The integration of ‘omics’ data, along with advancements in bioinformatics tools aided by AI and ML, has the potential to propel the field in an unprecedented manner [92,93]. However, such developments require truly interdisciplinary research efforts, which are currently limited to a select few top institutes. Addressing these challenges will be crucial in advancing the understanding and application of postbiotics in various fields.

## 3. Clinically Established Applications of Postbiotics

**Figure 5:**
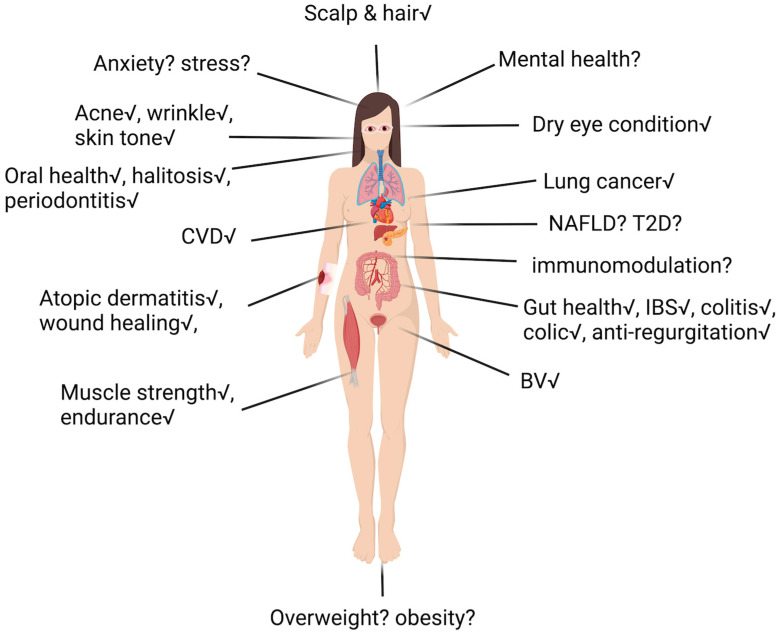
Established focus areas for proven clinical applications of postbiotics. A checkmark next to a condition indicates confirmed postbiotic efficacy, while a question mark implies ongoing investigation in clinical trials. This illustration was generated using the BioRender tool.

In light of the numerous recent reviews that have comprehensively outlined the overall physiological functions of postbiotics, our focus here is to delve into their specific proven clinical applications and their details (Figure 5). Clinical trials, primarily involving the digestive system followed by the integumentary system, are most commonly performed with postbiotic testing. The following section provides an up-to-date clinical trial update on postbiotics, and quite a few of these trials are not standalone; they also include potential prebiotics (see Table 2 for a summary). 

### 3.1. Clinical Application of Postbiotics in the Digestive System

Similar to probiotics and prebiotics, many trials with postbiotics are focused on applications related to digestive ailments. The following paragraphs briefly detail the type of trial, the ailment condition, the source of the postbiotic, and the outcomes of these trials.

Periodontitis, an inflammatory condition affecting both soft and hard tooth-supporting tissues, stands as a primary cause of tooth loss following dental decay. In a single-center, randomized controlled trial, the authors recruited individuals aged 18 to 70 with a history of periodontal disease [94]. The trial involved the application of a gel containing undisclosed postbiotics and other active constituents twice daily for 14 days. The effects of this postbiotic gel were compared with standard care, represented by the application of conventional chlorhexidine gel. The results indicated no significant differences between the postbiotic gel application and the conventional chlorhexidine gel application [94]. This suggests that postbiotics could serve as a natural alternative to conventional chlorhexidine therapy in addressing periodontitis, offering potential benefits for individuals with a history of periodontal disease.

In one study, authors compared the effects of heat-killed postbiotics and supernatant extract postbiotic from probiotic strains, namely *L. salivarius* subsp. *salicinius* AP-32, *L. paracasei* ET-66, and *L. plantarum* LPL28, known for their antipathogenic properties against oral pathogens [95]. Specifically, it examined the impact of lozenges containing heat-killed postbiotics from *L. paracasei* ET-66 and *L. salicinius* AP-32, along with supernatant postbiotics from all three strains. The participants enrolled in the study were individuals between 20 and 40 years. Both types of postbiotic lozenges demonstrated antibacterial activities, contributing to better oral health and improved intestinal conditions compared to the placebo group. Furthermore, the postbiotic groups exhibited increased levels of salivary immunoglobulin A and a higher population of beneficial oral bacteria, including *Lactobacillus* and *Bifidobacterium* [95]. Overall, the study revealed that regardless of the source of postbiotics, they effectively enhance oral immunity, inhibit the growth of oral pathogens, and increase the number of beneficial oral microbiota.

Halitosis, often linked to oral microbiome dysbiosis, was the focus of a randomized controlled trial. The trial aimed to assess the effects of postbiotic *L. paracasei* ET-22 heat-killed bacteria on halitosis and the associated oral microbiome [96]. Participants were instructed to apply lozenges containing the postbiotic three times daily for a duration of four weeks. The results revealed significant reductions in the pathogens responsible for halitosis, namely *Rothia* and *Streptococcus*, within the postbiotic group. Consequently, there was a notable decrease in the production of undesirable odors associated with halitosis in individuals receiving the postbiotic treatment [96]. This suggests the potential efficacy of postbiotic *L. paracasei* ET-22 in addressing halitosis and modulating the oral microbiome. 

In another randomized, double-blind, placebo-controlled clinical trial, the authors successfully showcased the transformative effects of a postbiotic derived from the probiotic strain *L. paracasei* ET22 [97]. This intervention notably altered the composition of total salivary VOCs, with a particular emphasis on aroma-active VOCs. Undesirable VOCs, including indole, pyridine, nonanoic acid, benzothiazole, and valeric acid, experienced a significant reduction in individuals with halitosis. Moreover, the study revealed a substantial inhibition of halitosis-associated pathogens like *Rothia* and *Streptococcus*. This suggests the clinical application of postbiotics in treating halitosis [97]. 

In a double-blind randomized control trial, the effects of a partly fermented infant formula enriched with postbiotics, including 2′-linked fucosyllactose and a specific prebiotic mixture of short-chain galacto-oligosaccharides and long-chain fructo-oligosaccharides, were investigated in healthy 14-day-old infants [12]. The study aimed to evaluate growth, safety, and tolerance in comparison to infants exclusively breastfed. The findings revealed no statistically significant differences in terms of weight gain, length, and head circumference between the breastfed group and the group fed with the postbiotic formula. Additionally, there were no reported adverse events or issues related to gastrointestinal tolerance [12]. These results suggest that the infant formula enriched with the tested postbiotics supports adequate infant growth and is safe and well tolerated in healthy term infants, comparable to infants exclusively breastfed. 

In a similar randomized, double-blind, placebo-controlled clinical trial, researchers compared an infant formula (Nutribén Innova 1, produced by Lacter Farmacia, Spain) supplemented with a thermally inactivated postbiotic (*Bifidobacterium animalis* subsp. *lactis*) to commercial infant formula (Nutribén Natal, produced by Lacter Farmacia, Spain) and breastfed infant groups [98]. The investigation focused on parameters such as weight gain, body composition, safety, and tolerability in infants less than 25 days old up to 6 and 12 months. The results indicated that both formula-fed and breastfed groups exhibited higher weight gain and BMI, with similar body composition, head circumference, and tricipital/subscapular skinfolds. Notably, the postbiotic-supplemented group showed consistent stool production and fewer respiratory, thoracic, and mediastinal disorders compared to the other two groups, indicating safety and tolerability [98]. In summary, while the postbiotic-supplemented formula was associated with lower weight and BMI compared to the commercial formula, it was found to be safe and well tolerated in infants, suggesting potential benefits in terms of gastrointestinal health and overall well-being.

In a double-blind placebo-controlled study on infants less than 91 days old, the authors tested a novel anti-regurgitation formula that contained postbiotics, short-chain galacto-oligosaccharides, and long-chain fructo-oligosaccharides [99,100]. The results indicate that the formula containing postbiotics is well tolerated, safe, and supports adequate growth. Importantly, it shows improvement in intestinal symptoms, especially in infants with more severe symptoms [99,100]. These findings suggest that the developed formula could be utilized as an anti-regurgitation formula in infants without causing any adverse effects.

In another study, the authors evaluated the effect of an infant formula containing postbiotics, specifically using the bacterial strains *Bifidobacterium breve* C50 and *Streptococcus thermophilus* 065, along with a specific prebiotic mixture (short-chain galacto-oligosaccharides and long-chain fructo-oligosaccharides). The study, conducted as a double-blind randomized control trial, aimed to assess the impact on the incidence of gastrointestinal symptoms, stool characteristics, sleeping and crying behavior, growth adequacy, and safety in infants less than 28 days old [101,102]. Two other groups were included for comparison: a non-postbiotic infant formula group and a breastfed control group. The results indicated no significant differences among the three groups in terms of daily weight gain and overall infant growth. No adverse events were reported in any of the tested groups. However, the postbiotic infant formula group exhibited a lower prevalence of infantile colic compared to the other two groups [101]. Overall, the postbiotic infant group supported infant growth, weight gain, and intestinal microbiota composition, and was well tolerated and safe, comparable to the breastfed group [102].

In a prospective multi-center clinical trial, researchers assessed the effect of postbiotic sodium butyrate on reducing the severity of clinical symptoms and improving the quality of life in patients with irritable bowel syndrome (IBS), aged over 18 years [103]. The study findings indicated a statistically significant improvement in the severity of abdominal pain, flatulence, diarrhea, constipation, urgent pressure for bowel movements, nausea, and vomiting in IBS patients who received the postbiotic [103]. This suggests that postbiotic sodium butyrate can be effectively used in managing the symptoms of IBS.

Researchers assessed the efficacy of the postbiotic product ReFerm^®^ (also known as Profermin^®^), which contains oat gruel fermented with *L. plantarum* 299v and is rich in SCFAs and other microbial metabolites [104]. This assessment was carried out in a randomized controlled trial involving individuals aged between 19 and 55 years who had mild-to-moderate IBS. The study findings revealed a significant improvement in intestinal barrier function in patients with IBS, emphasizing the potential benefits of the tested postbiotic in IBS management [104].

In a recent randomized double-blind, placebo-controlled trial, researchers evaluated the safety and efficacy of probiotic *B. longum* CECT 7347 and heat-treated postbiotic *B. longum* CECT 7347 in participants with diarrhea-predominant IBS [105]. The study recruited individuals aged 18 to 65 diagnosed with IBS-D. The findings indicate that both the probiotic and postbiotic groups were equally effective in reducing IBS symptom severity and improving stool consistency compared to the untreated control group. This study marks the first reported positive results for either a probiotic or postbiotic derived from the same strain in ameliorating IBS symptoms [105].

#### Promising Applications of Postbiotics in Gastrointestinal Animal Models

Since many of the postbiotic trials are being conducted with a special focus on improving GI disorders, the authors would like to list a few promising applications of postbiotics in GI animal models. Note that the authors have not given an in-depth discussion of the application of postbiotics in different animal species’ GI disorders, as this is beyond the scope of the current review. Readers interested in this aspect can refer to previously published recent articles for further information [106,107,108,109,110,111,112,113,114,115,116,117]. By highlighting these promising applications of postbiotics in GI animal models, the authors aim to underscore their potential therapeutic value in managing a range of GI disorders. However, further research is warranted to fully elucidate their mechanisms of action and optimize their clinical efficacy.

In a study, researchers utilized a dextran sodium sulfate-induced mouse model of ulcerative colitis (UC) to investigate the effects of probiotic *Bifidobacterium adolescentis* B8589 and its postbiotic (non-viable powder of *B. adolescentis* B8589) on symptoms, fecal microbiota, and their functional correlation [118]. Administration of the postbiotic and/or probiotic reduced disease activity index scores, ameliorated colon shortening induced by DSS, and significantly decreased inflammatory cell infiltration, mucosal damage, and crypt loss. Fecal microbiota analysis revealed a notable difference in microbiota structure only between the postbiotic and DSS groups, suggesting that postbiotic intervention was more effective than probiotics in restoring fecal microbiota diversity disrupted by DSS treatment. Furthermore, unlike the probiotic group, postbiotic-induced fecal microbiota showed a positive correlation with associated microbial metabolic pathways. Overall, these findings indicate that postbiotic administration can alleviate symptoms and pathophysiology associated with DSS-induced colitis and induce a more pronounced modulation effect on the fecal microbiome compared to probiotic administration [118].

In a recent similar DSS-induced colitis mouse model, researchers investigated the comparative effects of interventions with the probiotic *Saccharomyces boulardii* CCTCC NO.M 2012116 strain, a postbiotic (heat-killed *S. boulardii*), and a prebiotic (*S. boulardii* β-glucan) [119]. All three interventions equally restored body weight, reduced the disease activity index, inhibited splenomegaly, shortened colon length, and alleviated histopathological damage to colonic epithelial tissues in DSS-induced colitis mice. Furthermore, these treatments also increased the levels of tight junction proteins (Occludin and ZO-1) and decreased the levels of pro-inflammatory cytokines (TNF-α, IL-1β, and IL-6) in the serum and tissue. However, the impact on gut microbiota varied. The postbiotic, heat-killed *S. boulardii*, demonstrated maximal restoration of the composition, structure, and functionality of the intestinal microbiota to normal levels compared to the other two groups, suggesting that the heat-killed *S. boulardii* postbiotic has greater advantages over probiotic *S. boulardii* and prebiotic *S. boulardii* β-glucan in the treatment of intestinal disorders such as UC [119].

In another study, researchers investigated the effects of heat-killed *B. bifidum* B1628 in a DSS-induced colitis model [120]. Postbiotic treatment resulted in reduced disease severity, as evidenced by lower histology scores and decreased serum levels of pro-inflammatory cytokines (IL-1β and TNF-α), along with elevated levels of the anti-inflammatory cytokine IL-13 compared to the DSS group. Furthermore, postbiotic treatment ameliorated DSS-induced gut dysbiosis by increasing populations of beneficial bacteria such as *Lactobacillus* while decreasing levels of known pathogenic taxa in IBD, including *Porphyromonadaceae*, *Subdoligranulum*, *Lachnospiraceae bacterium* 3_1_46FAA, and *Alistipes indistinctus*. Functional metagenomics revealed three significantly enriched metabolic pathways in the postbiotic-treated group, namely the aerobic respiration I (cytochrome c) pathway, and the super pathways of L-phenylalanine biosynthesis and L-tryptophan biosynthesis, respectively. In conclusion, the study suggests that the heat-killed *B. bifidum* B1628 postbiotic effectively improves symptoms, inflammation, tissue damage, and gut dysbiosis in DSS-induced colitis mice [120].

In a rat model of periodontitis, periodontal disease was induced by placing a nylon thread ligature around the right mandibular first molars in each rat. The rats were then treated with a whey bioconversion product produced by *E. faecalis* M157 KACC 81148BP, and a combination of whey bioconversion products produced by *E. faecalis* and *L. lactis* ssp. *lactis* CAU2013 KACC 81152BP, with the aim of alleviating periodontitis (PD) and improving gut health [121]. Both postbiotics were orally administered to PD-induced rats for 8 weeks. The study observed a reduction in PD lesions in the group treated with the postbiotic *E. faecalis*, while no lesions were observed in the group treated with the mixture of *E. faecalis* and *L. lactis*. Additionally, both postbiotic groups showed reduced bone loss volumes and increased ratios of *Lactobacillus* spp. in the gut microbiome compared to the untreated control group. These findings suggest that the whey bioconversion product produced by *E. faecalis* and the mixed whey bioconversion products produced by *E. faecalis* and *L. lactis* are equally effective in relieving periodontitis and improving gut health [121]. 

Researchers investigated the impact of live and pasteurized postbiotic *Akkermansia muciniphila* on serum uric acid levels in a hyperuricemic mice model, exploring potential mechanisms involving changes in uric acid synthesis and excretion [122,123]. Mice received oral gavage for three weeks. Both forms of *A. muciniphila* administration reduced serum urate levels and inhibited xanthine oxidase in the liver, the primary enzyme responsible for uric acid generation. Furthermore, fecal and urinary urate levels increased in both treatment groups, aligning with elevated mRNA and protein expression of renal and intestinal uric acid-related transporters. Both bacterial forms also decreased mRNA expression of inflammatory factors in the liver, kidneys, and colon. Notably, only live *A. muciniphila* enhanced tight junction protein expression and ameliorated intestinal dysbiosis. These findings suggest that both live and pasteurized postbiotic *A. muciniphila* can effectively mitigate hyperuricemia by modulating uric acid metabolism and inflammation, with live bacteria exhibiting additional beneficial effects on gut microbiota [122].

Utilizing a high-fat-diet (HFD)-induced obese mouse model, researchers investigated the differences between the live and postbiotic (pasteurized) forms of *A. muciniphila* strains in their effects on lipid and glucose metabolism, as well as their regulation of immune responses [122,123]. The respective groups were administered either the live or pasteurized forms of two *A. muciniphila* strains five times per week for a period of 12 weeks. Both treatment groups demonstrated improvements in HFD-induced obesity and metabolic dysregulation, as evidenced by prevention of weight gain and a reduction in major adipose tissues, adipogenesis/lipogenesis, and serum cholesterol levels. These improvements were accompanied by physiological enhancements in glucose homeostasis and suppression of inflammatory responses. Furthermore, both treatment groups exhibited enhanced gut integrity and improved hepatic structure and function in HFD-induced animals. Of particular note, the postbiotic group demonstrated greater potency in improving glucose tolerance compared to the live form, suggesting that the postbiotic form of *A. muciniphila* has potential applications in reducing obesity [122,123].

### 3.2. Clinical Application of Postbiotics in the Integumentary System

To address the serious concern of age-associated muscle decline in older individuals, researchers conducted a randomized controlled trial involving participants aged 40 to 64 years [124]. The trial focused on the administration of two doses of the postbiotic urolithin A (UA) orally over a four-month period. UA, a well-known gut-microbiome-derived postbiotic, is produced through the metabolism of ellagitannins—polyphenolic compounds found in foods like pomegranates, berries, and walnuts. Pre-clinical models have demonstrated that the administration of UA induces mitophagy and enhances mitochondrial function, leading to improved muscle functions associated with aging [125,126]. In the clinical trial involving human participants, the authors observed significant improvements in endurance, physical performance, and reduced inflammation [124]. These findings strongly suggest the beneficial impact of postbiotic UA in enhancing muscle performance, providing a potential avenue for addressing age-related muscle decline in the elderly.

In a double-blind placebo-controlled clinical trial, researchers explored the wound-healing properties of the postbiotic *L. reuteri* lysate in individuals aged 19 to 42 years [127]. The study findings suggest that dietary supplementation with the postbiotic, a sterile lysate of this microbe, alone is sufficient to elevate systemic oxytocin levels. This elevation is correlated with a reduction in cortisol levels and an improvement in wound repair capacity in humans [127]. The results indicate that the postbiotic produced by *L. reuteri* may modulate host oxytocin secretion, holding potential implications for public or personalized health goals.

In a randomized double-blind controlled trial, researchers evaluated the effects of the postbiotic heat-killed *L. paracasei* MCC1849 on the physical condition of healthy adults across a wide age range (20 to 74 years) [128]. The postbiotic group exhibited a significant reduction in the number of days with symptoms such as stuffy nose, sore throat, and cold-like symptoms compared to the placebo group. Importantly, no side effects were observed in the postbiotic group [128]. These findings suggest that the postbiotic derived from the *L. paracasei* strain can be both safe and effective in suppressing symptoms, contributing to the maintenance of physical well-being in healthy individuals.

In a double-blind, placebo-controlled clinical trial, the effects of the postbiotic derived from heat-killed *L. plantarum* TWK10 were assessed on exercise endurance performance, muscle weight and strength, fatigue, and body composition in healthy males aged 20–40 years [129]. The group receiving the postbiotic demonstrated a significant increase in exercise endurance time and grip strength in both right and left hands compared to the control group [129]. These findings suggest that the postbiotic derived from the *L. plantarum* TWK10 strain holds significant potential as a physical performance enhancer for humans.

In a prospective randomized clinical study involving Asian women aged between 19 and 69 years, the researchers investigated the effects of a topically applied postbiotic *Epidermidibacterium Keratini* (EPI-7) ferment filtrate on skin aging [130]. The EPI-7 postbiotics, which include the metabolite orotic acid, were found to significantly enhance skin barrier function, improve skin elasticity, and restore commensal microbial diversity in the dermal layer. Crucially, the study reported no adverse effects during the research period, such as application-site itching, pain, or post-treatment erythema [130]. These findings suggest the potential of postbiotic therapy in cosmetics, highlighting the positive impact of EPI-7 postbiotics on addressing skin aging concerns without observable adverse reactions.

In a randomized controlled clinical trial involving Chinese females aged between 18 and 40 years, researchers investigated the effects of topical postbiotic cosmetic moisturizers [131]. Specifically, water gel moisturizers with and without yeast extract were compared to a commercial cream moisturizer. The study aimed to assess the impact on the skin and skin microbiome in the presence of environmental stressors. The results indicated that the postbiotic moisturizers, particularly those containing yeast extract, played a supportive role in regulating the skin’s microbiome. Furthermore, these postbiotic moisturizers were found to enhance the holistic skin barrier, involving the skin microbiome, physical, chemical, and immune barriers [131]. This comprehensive approach to skin protection against environmental stressors appeared more effective when compared to the commercial moisturizer used in the study. The findings suggest that yeast extract postbiotics have potential applications in cosmetics and may outperform existing commercial products in promoting skin health.

Previous research focused on assessing the impact of the heat-treated probiotic strain *Pediococcus acidilactici* LM1013 on inhibiting the growth and biofilm formation of *C. acnes* in individuals aged 14 to 40 years with acne vulgaris, conducted as a single-arm clinical study. The postbiotic ingredient effectively regulated the growth and biofilm formation of *C. acnes*. When applied as a cream for individuals with acne vulgaris, it exhibited the ability to diminish comedones and reduce facial sebum content. Notably, the majority of participants reported satisfaction with the cream containing postbiotics [131]. These findings suggest that postbiotic HT-LM1013 holds promise as a novel cosmetic ingredient for humans, demonstrating efficacy in addressing concerns related to acne vulgaris and promoting overall skin health.

Acne vulgaris, a chronic inflammatory skin disease, can have a lasting impact on one’s appearance if left untreated. Common acne therapies involve antibiotics, benzoyl peroxide, and azelaic acid. However, these medications often come with side effects and are not recommended for long-term use. In an innovative approach, the authors co-fermented collagen, known for its wound-healing properties, with three probiotic strains (TYCA06/AP-32/CP-9 or TAC) [132]. They then incorporated the fermented postbiotic material into a cosmetic gel, which was applied to subjects’ acne lesions in a randomized clinical trial. The postbiotic gel demonstrated significant antimicrobial activity against *Propionibacterium acnes*, the bacterium associated with acne, and exhibited anti-inflammatory effects around the lesion areas. The results included effective growth inhibition against *P. acnes*, reduced inflammation, and a decrease in the number of porphyrins and brown spots on facial skin [132]. This suggests that such postbiotics have beneficial effects on skin health and can ameliorate redness, inflammation, and acne symptoms in patients with acne vulgaris.

In a randomized double-blind placebo-controlled trial, researchers assessed the efficacy of rice flour containing heat-killed postbiotic *L. paracasei* CBA L74 on infants with moderate to severe atopic dermatitis (AD), aged 6–36 months [133]. Unfortunately, the postbiotic-treated group did not prove to be effective in significantly reducing the severity of AD in infants compared to conventional steroid therapy, which demonstrated a more pronounced effect. However, the postbiotic group exhibited a corticosteroid-sparing effect beyond the treatment period [133]. This suggests that the postbiotic could serve as an effective adjunctive treatment in AD management, potentially minimizing corticosteroid adverse effects and improving patients’ and parents’ adherence to therapy.

Healthy volunteers aged between 18 and 69 years were recruited to assess the moisturizing efficacy of a postbiotic cream [134]. The postbiotic, containing a blend of metabolites such as organic acids, enzymes, and peptides resulting from the co-fermentation of three probiotic strains (*L. plantarum* (AN057), *L. casei* (AN177), and *S. thermophilus* (AN157), was evaluated in a multicentric, randomized, intra-individual, double-blind group study. The application of the postbiotic cream resulted in increased skin moisture and elasticity, along with a reduction in pore size and wrinkle depth compared to the control [134]. These findings indicate that the postbiotic ingredient acts as a potential cosmetic agent, providing strong beneficial effects on human skin by enhancing moisture and elasticity while reducing wrinkles and pore size.

In a clinical trial involving individuals with an average age of 37, the beneficial effects of a shampoo containing heat-killed probiotics, specifically *L. paracasei* GMNL-653, were investigated [135]. The results indicated that the postbiotic-containing shampoo improved scalp conditions by effectively controlling sebum secretion, reducing dandruff generation, and promoting hair growth. Additionally, the shampoo was found to modulate the scalp microbiota. Overall, the findings suggest that the heat-killed *L. paracasei* GMNL-653 postbiotic-containing shampoo is not only equally effective but also surpasses the efficacy of certain aspects when compared to a commercial product [135]. This highlights the potential of postbiotic-containing shampoos in providing comprehensive benefits for scalp health and hair care.

In a randomized, double-blind, placebo-controlled study, the therapeutic effect and safety of tyndallized *L. rhamnosus* (IDCC 3201) were assessed in infants with AD aged between 1 and 2 years [136]. The postbiotic group showed an improvement in AD conditions with a decrease in inflammatory conditions. Additionally, the postbiotic treatment was well tolerated, and no adverse effects were recorded [136]. These findings suggest that postbiotics, such as tyndallized *L. rhamnosus*, can be used to effectively moderate atopic dermatitis in infants.

### 3.3. Clinical Application of Postbiotics in the Reproductive System

Vaginal dysbiosis is a primary cause of bacterial vaginosis (BV). In a clinical trial involving the intervention of a postbiotic, i.e., ferment of *L. paracasei* ProSci-92 and *L. rhamnosus* ProSci-109 in gel, the authors evaluated its efficacy in women diagnosed with BV, aged between 18 and 55 years [137]. The study findings indicated that the application of the postbiotic gel led to an improvement in BV symptoms and abnormal vaginal secretions. Moreover, it increased the relative abundance of beneficial lactobacilli in the vaginal microbiota while reducing the presence of potential pathogens such as *Gardnerella*, *Prevotella*, and *Atopobium*. Overall, the results demonstrated that the application of the postbiotic gel ameliorated BV, and this symptom improvement was associated with significant changes in the bacterial vaginal microbiota [137]. Hence, postbiotics could be a valuable supplement in clinical settings for effectively managing BV.

### 3.4. Clinical Application of Postbiotics in the Sensory System

In the context of dry eye disease (DED), the authors conducted a randomized, placebo-controlled clinical trial to assess the efficacy and safety of the postbiotic *Latilactobacillus sakei* [138]. The evaluation included two forms of administration: as an ophthalmic bacterial lysate (drops with no live organisms, i.e., postbiotic) and as an oral probiotic (live organisms). The trial involved individuals aged 16 to 60 years. The results demonstrated that the postbiotic ophthalmic formulation, containing *L. sakei* lysate, significantly improved both the signs and symptoms of DED. Additionally, it effectively suppressed the ocular surface inflammatory response. In contrast, the oral intake of *L. sakei* as a probiotic capsule had no discernible effect on these patients [138]. This suggests the potential application of postbiotics, particularly in the form of an ophthalmic solution containing *L. sakei* lysate locally, as a promising treatment for dry eye disease.

### 3.5. Clinical Application of Postbiotics in the Respiratory System

In a study, the author tested the postbiotic JK5G product prepared by Japan Kyowa Industrial Co., Ltd. (Sanjo City, Japan)—containing a high-concentration complex rich in bacteria and their metabolites, including *Lactococcus* and 21 kinds of compound lactobacillus bacteria, peptidoglycans, and metabolites from *Lactococcus cytoderm*—on small-cell lung cancer patients aged 40–70 undergoing chemotherapy in a randomized control trial [139]. The findings indicated that JK5G postbiotics played a role in attenuating immune-related adverse events, enhancing the quality of life, and improving gut microbiota. This, in turn, ameliorated the tumor microenvironment and reduced inflammation [139,140]. The study suggests that postbiotics can be a valuable therapeutic option when combined with existing therapies to manage cancer patients.

### 3.6. Clinical Application of Postbiotics in the Circulatory System

In a randomized controlled trial, the authors investigated the effects of enriched seafood sticks with a combination of postbiotic heat-inactivated *Bifidobacterium animalis* subsp. *lactis* CECT 8145, inulin, and omega-3 on cardiometabolic risk factors in abdominally obese subjects aged over 18 years [141]. The postbiotic-enriched seafood sticks demonstrated a potential protective effect against the development of type 2 diabetes. This was evidenced by a reduction in insulin resistance, lower atherogenic triglyceride postprandial concentrations, and improvements in cardiovascular disease risk factors [141]. Consequently, the findings suggest that postbiotic-enriched seafood sticks could serve as a complementary strategy in the management of cardiovascular disease.

**Table 2:** Description of the postbiotics under investigation, their source, the treatment regimen employed, the size of the clinical trial, the targeted condition, and the trial outcomes.

Postbiotic	Microbial Source	Dosage and Regimen	Trial Size	Subject’s Condition	Outcome
**Digestive system**					
Urolith	Gut microbes	500 and 1000 mg, oral twice daily, 4 months	88	Overweight but absence of any chronic medical	Improved muscle performance postbiotic treated compared to untreated control [124].
*Heat*-killed	*Lacticaseibacillus paracasei* *	Lozenges three times a day for 4 weeks	68	Halitosis	Significantly inhibited halitosis and improved oral microbiome [96].
Sodium butyrate	*Chemically synthesized*	Twice daily orally for 12 weeks	3000	Confirmed IBS patients	Effectively relieved the symptoms of IBS [103].
Heat-treated	*Bifidobacterium longum* CECT 7347	Once daily orally for 12 weeks	200	Diagnosed with IBS	Reduced IBS symptom severity [105].
Heat-killed and supernatant	*Ligilactobacillus salivarius* subsp. *salicinius* AP-32 *, *Lacticaseibacillus paracasei* ET-66 *, and *Lactiplantibacillus plantarum* LPL28 *	Oral lozenges thrice for 4 weeks	75	Healthy individuals	Enhanced oral immunity, inhibited oral pathogens, and increased beneficial oral microbiota [95].
2′-linked fucosyllactose	*Chemically synthesized*	Fed ad libitum orally until week 17	276	Healthy infants	Supported adequate infant growth and was well tolerated [12].
Thermally inactivated	*Bifidobacterium animalis* subsp. *lactis*, BPL1	Fed ad libitum orally up to 12 months	217	Healthy infants	Lowered BMI and found to be safe and well tolerated in infants [98].
Fermented formula	Lactofidus^TM^	Oral feeding for 4 weeks	182	Infants diagnosed with uncomplicated regurgitation	Improved symptoms, was well tolerated and safe [99,100].
Fermented formula fraction	Lactofidus^TM^, *Bifidobacterium breve* C50, and *Streptococcus thermophilus* 065	Fed ad libitum orally until week 17	200	Healthy infants less than 29 days	Supported infant development and was safe compared to the breastfed group [101].
Oat co-ferment	*Lactiplantibacillus plantarum* *	Twice-daily enema for 2 weeks	35	Patients diagnosed with moderate to severe IBS	Improved barrier-protective properties in IBS patients [104].
Heat-killed	*Lacticaseibacillus paracasei* MCC1849 *	Orally once daily for 24 weeks	586	Healthy individuals	Suppressed subjective symptoms in healthy adults [128].
Heat-killed	*Lactiplantibacillus plantarum* TWK10 *	Twice daily orally for 6 weeks	30	Healthy individuals	Significantly improved endurance [129].
Ferment	*Bifidobacterium breve* C50 (BbC50) and *Streptococcus thermophilus* ST065	Ad libitum oral feeding for 6 months	280	Healthy full-term infants	Normal growth, postbiotic was well tolerated, and microbiome composition and metabolic activity were similar to those of breastfed infants [141].
**Integument system**					
Ferment filtrate	*Epidermidibacterium Keratini* (EPI-7)	Facial application twice daily for 3 weeks	55	Healthy women	Significantly enhanced skin tone and skin microbiome diversity [130].
Yeast extract	*Pichia anomala*	Facial application twice daily for 8 weeks	110	Healthy women	Enhanced skin barrier-protective function and microbiome composition [131].
Heat-killed	*Lacticaseibacillus paracasei*, GMNL-653 *	Once- or twice-daily hair wash for 4 months	22	Healthy adults	Improved scalp conditions by controlling sebum secretion and dandruff generation, and promoting hair growth [135].
Lysate	*Limosilactobacillus reuteri* ATCC-PTA-6475 *	Consumed twice daily for 3 weeks	14	Healthy females following wounding by biopsy	Improved wound repair [127].
Heat-treated	*Pediococcus acidilactici* LM1013	Dosage information not available	23	Patients diagnosed with acne vulgaris	Inhibited acne vulgaris.
Collagen co-ferment	*Lactobacillus acidophilus TYCA06* *, *Ligilactobacillus salivarius* AP-32, and *Bifidobacterium animalis* subsp. *lactis* CP-9	Applied twice daily for 4 weeks	20	Patients diagnosed with acne vulgaris	Ameliorated redness, inflammation, and acne symptoms [132].
Rice-flour co-ferment	*Lacticaseibacillus paracasei* CBA L74 *	Once daily orally for 12 weeks	50	Infants and kids diagnosed with AD	Not effective in reducing the severity of AD but showed a steroid-sparing effect [133].
Metabolites including lipoteichoic acid, hyaluronic acid, lactic acid, and sphingomyelinase	*Lactobacillus plantarum* (AN057) *, *Lacticaseibacillus casei* * (AN177), and *Streptococcus thermophilus* (AN157)	Twice-daily application on skin for 4 weeks	50	Healthy individuals with no prior skin conditions	Significant beneficial effects on skin, with reduction in wrinkle depth and pore size [134].
Tyndallized extract	*Lacticaseibacillus rhamnosus* * (IDCC 3201)	Applied twice daily for 12 weeks	100	Infants with diagnosed AD conditions	Improved AD and reduced inflammation [136].
**Reproductive system**					
Ferment	*Lacticaseibacillus paracasei* * ProSci-92 and *L. rhamnosus* ProSci-109	Application in deep part of vagina every night for 7 days	50	Diagnosed with bacterial vaginosis (BV)	Ameliorated BV conditions and the symptom [137].
**Sensory system**					
Bacterial lysate	*Latilactobacillussakei* *	1 drop in each eye every 5 h for 4 weeks	40	Patients with dry eye syndrome	Significantly improved the signs and symptoms of DED and suppressed ocular inflammation [138].
**Respiratory system**					
Complex metabolites	21 kinds of lactobacillus not disclosed	Consumed orally on day 1 of each of the four 3-week treatment cycles	60	Patients confirmed with non-small-cell lung cancer	Attenuated the tumor microenvironment and inflammation [139,140].
**Circulatory system**					
Heat-inactivated	*B. animalis* subsp. *lactis* CECT 8145	Daily 50 g ingestion for 12 weeks	120	Abdominally obese individuals	Improved insulin resistance, circulating triglyceride levels, and risk factors for cardiovascular diseases [141].

* The genus name “*Lactobacillus*” has been amended as per a recent reclassification effort proposed by Zhang et al. [69].

### 3.7. Ongoing Extended Clinical Application of Postbiotics

The positive outcomes observed in human clinical trials across various systems have ignited a burgeoning interest among researchers to explore and expand the application space of postbiotics. Ongoing and completed trials (Table 3) are venturing into novel areas such as stress and anxiety management, addressing conditions like obesity, type 2 diabetes, overweight, and non-alcoholic liver disease. Additionally, postbiotics are being investigated for their potential to enhance mental health, anti-aging strategies, and immune modulation. The anticipation of favorable results from these trials holds the promise of significantly advancing the field, garnering increased attention and support from researchers, physicians, and pharmaceutical interests alike. This marks an exciting era where the multifaceted benefits of postbiotics may revolutionize healthcare and well-being. 

However, it is important to note that the advocacy of postbiotics in healthy individuals can be debated. For those who maintain a balanced diet rich in fiber and low in processed carbohydrates, coupled with physical and mental fitness, the direct consumption of postbiotics may offer little additional benefit, as the gut already produces ample amounts of postbiotics naturally. Conversely, if the diet lacks the necessary balance to support postbiotic production, the use of postbiotic supplements becomes a more logical choice. 

### 3.8. Conclusions

There is a significant surge of interest in postbiotics owing to numerous associated merits in comparison to probiotics. While many postbiotics trials have exhibited promising outcomes, several concerns warrant attention. Firstly, numerous postbiotic clinical trials lack adequate controls to precisely assess their effects. Often, postbiotics are combined with other ingredients or tested without matching postbiotic and probiotic effects, thus complicating interpretation. Secondly, trials have often utilized either crude complex postbiotic mixtures or well-known postbiotics, limiting the exploration of novel postbiotics. Thirdly, the discovery and characterization of new postbiotics present challenges that necessitate an interdisciplinary approach. Fourthly, ensuring the quality control of postbiotics is essential. Similar to probiotics, quality control regulations should not only ascertain the absence of contaminants but also ensure precise correspondence between label claims and actual contents, including the number of live cells, species, and strains. Additionally, stringent parameters need to be enforced for postbiotics, considering the added complexity of evaluating and controlling the types and amounts of contained molecules. Addressing these concerns is imperative to propel the field of postbiotics forward. 

## Figures and Tables

**Table 3:** Ongoing and completed clinical applications of postbiotics, specifying the targeted conditions and including active links to trial details for further exploration.

Postbiotic Trial Application	Study Description	Link
Anxiety	Evaluate the efficacy of a multistrain postbiotic administration for moderate anxiety in adults aged between 18 and 65 years.	https://clinicaltrials.gov/study/NCT05562739?intr=Postbiotic&limit=25&page=1&rank=1 (accessed on 28 August 2024) https://clinicaltrials.gov/study/NCT05562752?intr=Postbiotic&limit=25&page=1&rank=12 (accessed on 28 August 2024)
Stress	Evaluates the effects of colonic delivery of a postbiotic on stress response, mood state, sleep, and cognition in healthy young subjects aged between 21 and 30 years.	https://clinicaltrials.gov/study/NCT06097182?intr=Postbiotic&limit=25&page=1&rank=2 (accessed on 28 August 2024)
Weight	Evaluate the efficacy of a postbiotic supplement on weight management and metabolic health in individuals aged 18 and above with a BMI ranging between 25 and 32.	https://clinicaltrials.gov/study/NCT05912699?intr=Postbiotic&limit=25&page=1&rank=3 (accessed on 28 August 2024) https://clinicaltrials.gov/study/NCT05428137?intr=Postbiotic&limit=25&page=1&rank=5 (accessed on 28 August 2024)
Obesity	Investigate the effect of a postbiotic on obesity in individuals aged 18 to 65 years with a BMI greater than 25	https://clinicaltrials.gov/study/NCT05440630?intr=Postbiotic&limit=25&page=1&rank=4 (accessed on 28 August 2024) https://clinicaltrials.gov/study/NCT04151823?intr=Postbiotic&limit=25&page=1&rank=16 (accessed on 28 August 2024)
NAFLD	Assesses the short-term efficacy and safety of postbiotics in patients with NAFLD aged 18 to 70 years.	https://clinicaltrials.gov/study/NCT05804422?intr=Postbiotic&limit=25&page=1&rank=6 (accessed on 28 August 2024)
Type2 Diabetes	Assess the short-term efficacy and safety of postbiotics as an adjunct to standard anti-diabetic therapy in type 2 diabetic patients aged 18 to 70 years with HbA1c levels between 6.5 and 10.	https://clinicaltrials.gov/study/NCT05770076?intr=Postbiotic&limit=25&page=1&rank=7 (accessed on 28 August 2024) https://clinicaltrials.gov/study/NCT04639492?intr=Postbiotic&limit=25&page=1&rank=10 (accessed on 28 August 2024)
IBS	Investigates the beneficial effects of postbiotics on intestinal epithelial barrier function in patients with IBS.	https://clinicaltrials.gov/study/NCT05475314?intr=Postbiotic&limit=25&page=1&rank=8 (accessed on 28 August 2024)
Intranasal safety	Safety evaluation of intranasal postbiotic use in healthy volunteers aged 18 years and older.	https://clinicaltrials.gov/study/NCT05984004?intr=Postbiotic&limit=25&page=1&rank=9 (accessed on 28 August 2024)
Infant formula	Evaluates the effect of infant formula supplemented with postbiotics on the metabolome profiles of late preterm infants.	https://clinicaltrials.gov/study/NCT06052592?intr=Postbiotic&limit=25&page=1&rank=11 (accessed on 28 August 2024)
Mental health	Evaluate the effect of postbiotics on cognitive skills in healthy individuals aged 18 to 32 years.	https://clinicaltrials.gov/study/NCT04324749?intr=Postbiotic&limit=25&page=1&rank=13 (accessed on 28 August 2024) https://clinicaltrials.gov/study/NCT05738746?intr=Postbiotic&limit=25&page=1&rank=14 (accessed on 28 August 2024)
Muscle strength	Evaluates the efficacy and safety of postbiotic supplementation in patients aged 50 years and older with macular degeneration.	https://clinicaltrials.gov/study/NCT05056025?intr=Postbiotic&limit=25&page=1&rank=15 (accessed on 28 August 2024)
Aging	Assesses the efficacy and tolerance of postbiotics in an anti-aging daily serum for individuals aged 34 to 60 years.	https://clinicaltrials.gov/study/NCT05514782?intr=Postbiotic&limit=25&page=1&rank=17 (accessed on 28 August 2024)
Immunomodulation	Evaluates the immune-modulating effect of postbiotics in healthy individuals aged 18 to 30 years.	https://clinicaltrials.gov/study/NCT05819424?intr=Postbiotic&limit=25&page=1&rank=18 (accessed on 28 August 2024)

NAFLD stands for non-alcoholic liver disease, and IBS stands for irritable bowel syndrome.

## Data Availability

No new data were created or analyzed in this study. Data sharing is not applicable to this article.
